# Complications of use of vascular devices during the covid-19 pandemic: a retrospective cohort

**DOI:** 10.1590/1677-5449.202400772

**Published:** 2025-05-19

**Authors:** Johann Viktor Müller, Wander Eduardo Sardinha, José Manoel da Silva Silvestre, Guilherme da Silva Silvestre, Ualid Saleh Hatoum, Natália Scaneiro Boy Sardinha, Renne Rodrigues, Mariana Ragassi Urbano

**Affiliations:** 1 Universidade Estadual de Londrina – UEL, Hospital Universitário – HU, Londrina, PR, Brasil.; 2 Universidade Federal da Fronteira Sul, Chapecó, SC, Brasil.; 3 Universidade Estadual de Londrina – UEL, Departamento de Estatística, Londrina, PR, Brasil.

**Keywords:** intraoperative complications, vascular surgical procedures, vascular access devices, foreign bodies, complicações intraoperatórias, procedimentos cirúrgicos vasculares, dispositivos de acesso vascular, corpos estranhos

## Abstract

**Background:**

During the COVID-19 pandemic, there was a substantial increase in the number of severely ill hospitalized patients, which coincided with a corresponding increase in the consumption of medical supplies, including vascular devices. In this context, vascular surgeons perceived an absolute increase in complications associated with their use.

**Objectives:**

To calculate the rate of severe complications requiring surgical vascular intervention following vascular device implantation during the COVID-19 pandemic.

**Methods:**

A retrospective cohort was conducted to investigate complications associated with vascular devices, such as central venous catheters (CVC), arterial lines, peripherally inserted central catheters (PICCs), totally implantable venous catheters, and semi-implantable venous catheters. The exposed population was defined based on the number of vascular devices used during the pandemic period, identified using the WPDHOSP materials management software. A total of 1,708 consultations with the vascular surgery team were analyzed using Medview medical record software. Patient records were evaluated, selecting those requiring vascular intervention.

**Results:**

Out of a total of 16,988 vascular devices used, 25 patients needed surgical or endovascular vascular interventions. This corresponds to a severe complication rate of 0.14%. The complications found were intravascular foreign body, active bleeding, pseudoaneurysm, unintentional arterial implantation, expanding cervical hematoma, acute limb ischemia, and arteriovenous fistula. Patients underwent vascular procedures such as foreign body removal, arterial repair, arterial embolization, endovascular stenting, arterial thrombectomy, and arteriovenous fistula repair.

**Conclusions:**

The severe complication rate is consistent with incidences found in the pre-pandemic literature.

## Resumo

**Contexto:**

Durante a pandemia de covid-19, houve um aumento substancial no número de pacientes gravemente hospitalizados, que coincidiu com um aumento correspondente no consumo de insumos médicos, inclusive de dispositivos vasculares. Nesse contexto, os cirurgiões vasculares perceberam um aumento nas complicações associadas ao uso desses dispositivos.

**Objetivos:**

Calcular a taxa de complicações graves que requerem intervenção vascular após o implante de dispositivos vasculares durante a pandemia de covid-19.

**Métodos:**

Foi conduzido um estudo de coorte retrospectivo para investigar complicações associadas a dispositivos vasculares, como cateteres venosos centrais, linhas arteriais, cateteres centrais de inserção periférica, cateteres venosos totalmente implantáveis e cateteres venosos semi-implantáveis. A população exposta foi definida com base no número de dispositivos utilizados durante o período da pandemia, identificado por meio do *software* de gestão de materiais WPDHOSP. Um total de 1.708 consultas da equipe de cirurgia vascular foi analisado usando o *software* de prontuário médico Medview. Os prontuários dos pacientes foram avaliados, incluindo aqueles que necessitaram de intervenção cirúrgica vascular.

**Resultados:**

Vinte e cinco pacientes necessitaram de intervenção cirúrgica ou endovascular de um total de 16.988 dispositivos utilizados. Isso corresponde a uma taxa de complicações graves de 0,14%. As complicações encontradas foram corpo estranho intravascular, sangramento ativo, pseudoaneurisma, implante arterial inadvertido, hematoma cervical expansivo, isquemia aguda de membro e fístula arteriovenosa. Os pacientes foram submetidos a procedimentos vasculares como retirada de corpo estranho, reparo arterial, embolização arterial, colocação de stent endovascular, trombectomia arterial e reparo de fístula arteriovenosa.

**Conclusões:**

A taxa de complicações graves é consistente com as incidências encontradas na literatura pré-pandemia.

## INTRODUCTION

Use of devices for vascular access, particularly in the venous system, is widely practiced in hospital settings. During the COVID-19 pandemic, referral hospitals treating patients with COVID increased the number of Intensive Care Unit (ICU) beds^1^ and, consequently, their use of such devices.^2^

Although these devices are essential for the care of critically ill patients, their use and implantation can lead to complications, with the most common described in literature being catheter implantation failure (22.3%), arterial puncture (4.7%), catheter misplacement (3.6%), pneumothorax (1.3%), subcutaneous hematoma (0.8%), hemothorax (0.3%), and death (0.3%).^3^ Despite the existence of safety routines for patients, it is not known what impact the COVID-19 pandemic has had on the rates of these complications. This is why this study aimed to investigate the rate of severe vascular complications necessitating surgical or endovascular intervention in a tertiary hospital in southern Brazil during the COVID-19 pandemic. Additionally, the various types of injuries identified were discussed, along with the treatment methods utilized, and our findings were correlated with the existing literature.

## METHODS

### Study design and context

An analytical, observational, longitudinal study, classified as a retrospective cohort study was conducted at a single center, investigating the number of venous and arterial vascular devices and their severe mechanical complications within the vascular system, specifically those requiring conventional or endovascular surgical intervention. Given that the outcome of interest is rare, a census study, which is more appropriate to the study objective,^4,5^ was carried out covering the period from March 2020 to April 2022. An ideal sample size calculation is not therefore applicable.^6^ This period was recognized by the Ministry of Health as a Public Health Emergency of National Importance due to the COVID-19 pandemic. Data were collected from September 4th to September 15th, 2023, at the University Hospital of the State University of Londrina (HU-UEL) facilities.

The HU-UEL served as a referral hospital for the most complex cases of COVID-19 and increased its total number of beds from 294 to 465. Prior to the pandemic, there had been 41 ICU beds (including adult, pediatric, neonatal, and a burns unit), but at the peak of the pandemic, the hospital expanded capacity to 158 ICU beds.

### Participants

The survey of patients affected by vascular complications was conducted by analyzing consultation requests for the vascular surgery team for patients admitted to HU-UEL, using the electronic medical records software Medview. A review was conducted of all consultation requests during the pandemic period and all patients with consultation requests related to use of vascular devices were initially considered eligible. Complications from interventional radiological, cardiac, neurological, and vascular hemodynamic procedures were excluded. It is important to point out that fitting of vascular devices, such as double-lumen venous catheters, triple-lumen catheters, peripherally inserted central catheters (PICCs), and arterial lines, is not exclusively performed by the vascular surgery team at the HU-UEL, since other specialties also perform these tasks because of the high demand for use and replacement of these devices.

Following this initial selection, a second screening was conducted applying additional exclusion criteria. Requests for device implantation, evaluation of infected devices, non-surgical complications of vascular devices, and complications treated non-surgically were excluded. Among the final list of patients, those with severe mechanical complications were identified. These patients were assessed for demographic profile and COVID-19 diagnosis and subdivided into 7 types of complication: intravascular foreign body, active bleeding, pseudoaneurysm, unintentional arterial implantation, expanding cervical hematoma, acute limb ischemia, and arteriovenous fistula. Regarding COVID-19 status, patients were defined as confirmed cases if they exhibited symptoms and had a positive laboratory test for the disease. It is noteworthy that the HU-UEL confirmed cases of COVID-19 using duly validated tests and was one of the first centers in the country to use the molecular biology test and later used rapid tests.

The population exposed was determined by assessing the number of catheters utilized during the pandemic period using the WPDHOSP software’s ESTHOS inventory control module at the Materials Department of the HU-UEL. Each type of catheter used, including double-lumen central venous catheters (CVC), triple-lumen central venous catheters, arterial lines, semi-implantable venous catheters, totally implantable venous catheters, and PICCs, was counted individually, even if more than one device was used in the same patient. Each catheter replacement or implantation in a new location was considered a new exposure to the risk of developing iatrogenic injury. All sizes of devices were included, covering both adult and pediatric populations. It was not feasible to subdivide the sample into adult and pediatric groups, since catheters were not exclusively used within their respective target populations. This could introduce selection bias, as some adults may have had pediatric catheters fitted, particularly for invasive blood pressure monitoring when arteriotomy kits were unavailable, and older children may have had adult-sized catheters fitted. Additionally, since PICCs are utilized in both populations, this further increases the potential for selection bias as previously described. Simple peripheral intravenous devices were not included in the study.

### Statistical analysis

For the variable “Implanted Vascular Devices”, frequencies during the period (from March 2020 to April 2022) were examined according to the type of device and their respective daily consumption averages. Data on patient characteristics include mean age and frequencies of the variables sex (Female and Male) and COVID-19 (confirmed and not confirmed). For the variables “clinical data of patients undergoing intervention” and “intravascular foreign body”, frequencies of the categories for each variable are provided. The data were evaluated in terms of absolute (n) and relative frequency (%), dispersion measures for continuous data (mean, median, and mode), with calculation of the complication incidence rate and the 95% confidence interval (CI 95%) and shown in the Results section. The data were stratified by adding the PICC subpopulation variable. The rate of severe complications was calculated based on the frequency of PICC use among patients who underwent interventions involving catheters from this subpopulation.

### Ethical considerations and compliance

The study was conducted at the University Hospital of the State University of Londrina (HU-UEL). Authorization to conduct the study, which involved data and medical records research, was obtained from the Superintendent Board of HU-UEL. The study project was submitted to the Ethics Committee of the State University of Londrina, receiving the CAEE registration number: 72864223.3.0000.5231, and approval was granted with registration number: 6282193.

To guide our research, we used the Strengthening the Reporting of Observational Studies in Epidemiology (STROBE) Statement for Cohort Studies.

## RESULTS

**Figure 1:**
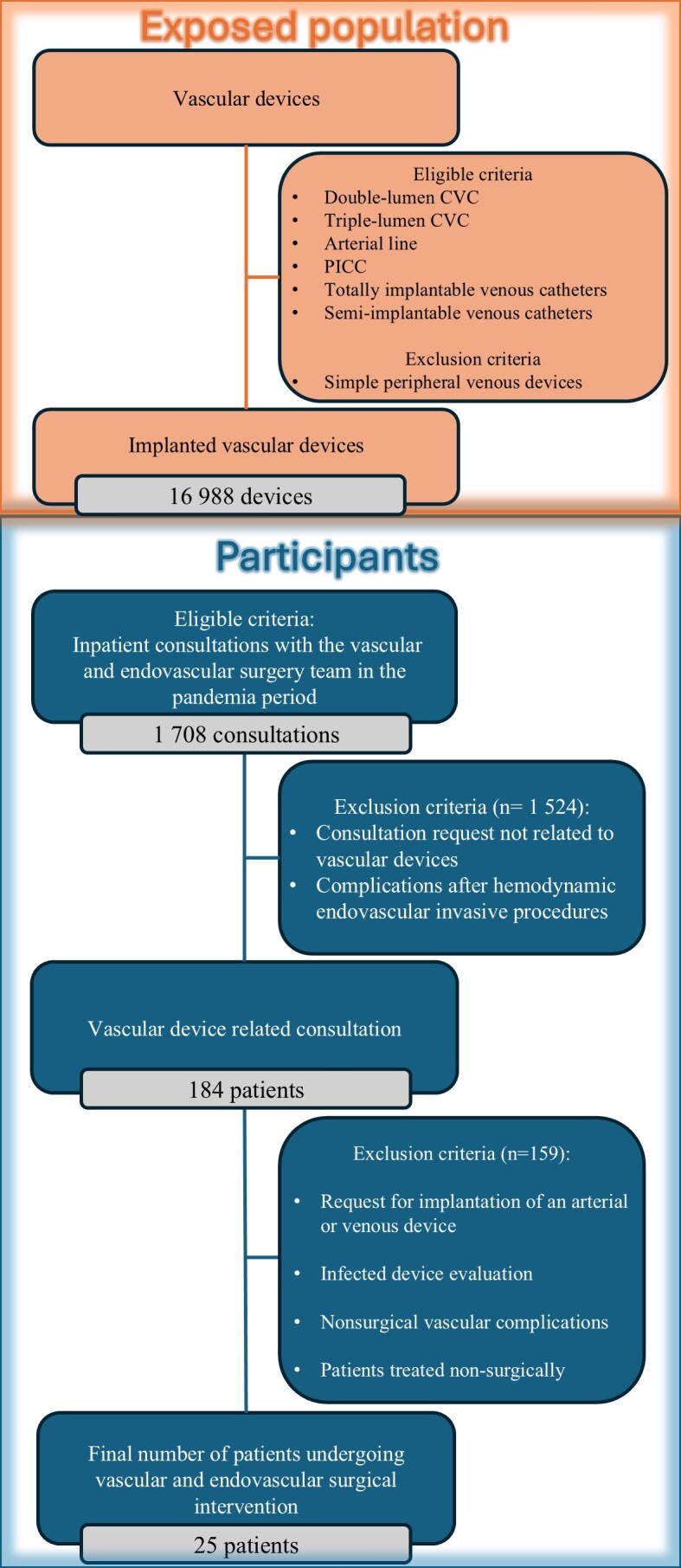
Flowchart of exposed population (orange) and patient inclusion (blue).

During the period, an average of almost 22 catheters were used per day, totaling 16,988 catheters, classified as shown in Table 1. A summary of the participants included in the study can be found in Figure 1. Since the research is a retrospective cohort study based on electronic medical records, there were no losses to follow-up or death that compromised collection of data on the patients included in the study.

**Table 1:** Vascular devices implanted and average number of implantations per day during the period from March 2020 to April 2022.

**Material**	**Implants**	**Daily average**
**Adult 6/7F double lumen central venous catheter (CVC)**	7 554	9.55
**Invasive arterial pressure kit**	4 682	6.14
**Double lumen 12F x 20cm for adult hemodialysis**	2 187	2.76
**Triple lumen 12F x 20cm for adult hemodialysis**	812	1.03
**Peripherally Inserted Central Catheter - PICC**	641	0.81
**Double lumen 11.5F x 16cm for adult hemodialysis**	541	0.68
**Double lumen 3/4F CVC for pediatric use**	400	0.52
**Double lumen 5/6F CVC for pediatric use**	140	0.18
**Totally implantable long-term catheter**	20	0.03
**Catheter 8/9/10F for pediatric hemodialysis**	9	0.01
**Semi-implantable long-term catheter**	2	< 0.01

Among the 25 patients with complications related to vascular devices, age ranged from 1 to 85, with a mean of 47.24 years, a median of 55 years, and the mode was 76 years, with 3 cases. A summary of patient information is shown in Table 2.

**Table 2:** Clinical data of patients undergoing intervention (n=25).

Age, mean ± standard deviation	47.24 ± 9.91
Sex, n (%)	
Female	16 (64)
Male	9 (36)
COVID, n (%)	
Not confirmed	17 (68)
Confirmed	8 (32)

The overall rate of severe mechanical complications requiring vascular or endovascular surgery after use of vascular devices in the service was 0.14% during the pandemic period (25 patients out of 16 988 devices) (95% CI 0.09% to 0.21%).

### PICC subgroup

The stratified rate of severe mechanical complications requiring vascular or endovascular surgery after use of a PICC was 0.31% during the pandemic period (2 patients out of 641 devices) (95% CI 0.05% to 1.25%). One patient underwent endovascular snare removal of a PICC fragment that had embolized to the pulmonary artery. The other patient required cervical subcutaneous exploration, including ligation of the external jugular vein and removal of the PICC, due to a knot at the PICC tip that prevented removal by standard manual traction. Both patients tested negative for COVID-19 and exhibited no clinical abnormalities beyond these catheter-related complications.

### Clinical data

We observed seven types of complication requiring surgical intervention, and their clinical presentations and forms are summarized in Table 3.

**Table 3:** Complications of patients who underwent interventions (n=25).

**Complication**	**n (%)**
Intravascular foreign body	10 (40)
Active bleeding	5 (20)
Pseudoaneurysm	4 (16)
Unintentional arterial implantation	3 (12)
Expanding cervical hematoma	1 (4)
Acute limb ischemia	1 (4)
Arteriovenous fistula	1 (4)

### Intravascular foreign body

Cases of intravascular foreign bodies presented the following characteristics: the material most often found was fragments of totally implantable catheters, followed by puncture wires from the kit, and fragments of PICC catheters. Most cases were treated by endovascular removal of the material using a snare catheter (Table 4).

**Table 4:** Summary of findings of intravascular foreign bodies (n=10).

**Location**	**n (%)**
Vena cava	5 (50)
Pulmonary artery	2 (20)
Right atrium	1 (10)
External jugular vein	1 (10)
Internal jugular vein	1 (10)
**Material Involved**	**n (%)**
Totally implantable catheter fragment	4 (40)
Puncture kit guidewire	3 (30)
PICC catheter fragment	2 (20)
Fragment of hydrophilic guidewire	1 (10)
**Treatment**	**n (%)**
Endovascular removal with snare catheter	8 (80)
Open surgical exploration	2 (20)

In two cases, endovascular treatment was not possible. In the first case, the patient had central stenosis and superior vena cava syndrome, leading to the decision to undertake surgical exploration and removal of a fragment of hydrophilic guidewire from the internal jugular vein. The second case was described above, in the PICC subgroup section.

### Active bleeding

The 5 cases of massive active bleeding had the following characteristics: all were implanted in the femoral artery. Four of these cases were hemodialysis catheters that were inadvertently implanted and removed by the treating clinician, and then active bleeding began. The average removal time after implantation was 4.06 days (8 hours, 1 day, 2 days, 6 days, and 11 days).

In one case, a double lumen 7F catheter was intentionally implanted in the femoral artery for hemodynamic monitoring of a severely shocked patient who had no other available site for invasive pressure monitoring. This catheter remained in place for 6 days until removal.

All patients were treated with surgical exploration and emergency vessel repair.

### Pseudoaneurysm

Four cases of pseudoaneurysm were diagnosed following implantation or attempted implantation of venous or arterial catheters. In all cases, our initial strategy involved ultrasound-guided local compression, along with correction of any blood dyscrasias and suspension of anticoagulant therapy. However, satisfactory results were not achieved despite these measures, necessitating additional interventions, either surgical or endovascular.

In two cases, there was no associated material, and no catheter was implanted at the site; only multiple attempts at puncture in the inguinal region for catheter implantation occurred. The vessels which developed pseudoaneurysms were the inferior epigastric artery and the lateral femoral circumflex artery, both of which were treated with coil embolization. In the other two cases, pseudoaneurysms of the femoral artery were diagnosed after use of hemodialysis catheters that had already been removed by the treating clinician.

### Unintentional arterial implantation

In our series, all cases of unintentional arterial implantation requiring open surgical or endovascular intervention involved hemodialysis catheters (11.5/12F). Two cases were treated with endovascular placement of a covered stent, because they were in the subclavian artery. The third case involved implantation in the internal carotid artery and was treated with surgical exploration and arterial repair with simple suture.

### Expanding cervical hematoma

The case of expanding cervical hematoma occurred after inadvertent implantation of a hemodialysis catheter in the common carotid artery. The catheter was removed a few minutes later by the medical team which performed the implantation. Emergency cervical exploration with arterial repair was required.

### Acute limb ischemia

One case presented with acute limb ischemia (Rutherford 2b) in the left lower limb 2 hours after inadvertent implantation of a hemodialysis catheter (12F) in the common femoral artery. Treatment involved surgical removal of the catheter, Fogarty thrombectomy, and arterial repair.

### Arteriovenous fistula

A pediatric patient developed a hematoma in the right inguinal region after removal of a 10F hemodialysis catheter used for 19 days. Upon evaluation, we identified an arteriovenous fistula between the femoral artery and femoral vein, requiring surgical treatment for repair.

## DISCUSSION

When reviewing the literature, some articles correlating the pandemic with complications of vascular devices were found. In a retrospective cohort, the percentage of patients who experienced a catheter-related complication was higher in the COVID-19 group compared to the non-COVID-19. However, this difference did not attain statistical significance (p = 0.057).^7^ Gidaro et al. (2022)^8^ reported that complications such as peri-catheter venous thrombosis, catheter-related bloodstream infection, and inadvertent catheter removal were significantly more frequent in COVID-19 patients. In our series, only 32% of patients with severe mechanical complications had a confirmed diagnosis of COVID-19 and one limitation of our study is the inability to separate and analyze the 16,988 devices used between COVID-19 and non-COVID-19 groups in our database.

We found a 0.14% rate of severe mechanical vascular complications from device use, similar to literature reports from pre-pandemic periods. According to Parienti et al. (2015)^9^, mechanical complications associated with catheter implantation had incidence rates for subclavian, jugular, and femoral sites of 2.1%, 1.4% and 0.7%, respectively. In a prospective cohort study at a teaching hospital published in the J Vasc Bras. by Jatczak et al.,^10^ a 16.44% incidence of mechanical injuries was reported. Other studies indicate that severe mechanical complications, including pneumothorax, massive bleeding, and death, occur with rates varying from 0.2%^11^ to 1.9%.^12^

In the PICC subgroup, severe mechanical vascular complications were found in 0.31% of patients. A review of the literature confirms that PICC fracture and embolization are rare occurrences, observed in less than 1% of cases,^13^ aligning with the findings of this study. Although cases of PICC knotting are reported, no incidence data is available and individualized treatment is essential.^14^

In regard to the cases of intravascular foreign bodies, all were successfully removed without any additional complications. The prompt recognition of the complication and its resolution contributed to the success rate, along with the minimally invasive endovascular techniques used in most patients, consistent with findings in other articles.^15-18^ The literature describes various complications related to intravascular foreign bodies, such as pulmonary embolism, endocarditis, and cardiac arrhythmias.^19-21^ A complication rate of 71% has been reported after embolism of unretrieved foreign objects,^22^ and this situation is associated with a high mortality rate of 23.7% when left unretrieved.^23^

Regarding the active bleeding events identified in our study, all patients underwent emergency surgery. Significant bleeding or hematoma after catheter removal and inguinal compression occurs in 1.0% to 2.4% of patients^24^ and catheter diameter, dwell time, and insertion site are risk factors for severe complications.^25^ The incidence of accidental arterial puncture during attempts at central venous catheterization is estimated at approximately 5%.^2^ In our cases of pseudoaneurysm, where 2 out of 4 patients did not receive implantable devices, we observed that multiple puncture attempts were identified as the primary cause of the pseudoaneurysms. These cases were treated with coil embolization. It is crucial to consider viable alternatives for pseudoaneurysm treatment. Previous research has emphasized the efficacy of injectable thrombin for managing pseudoaneurysms resulting from arterial catheterization procedures.^26,27^

In the remaining two cases, pseudoaneurysms located in the femoral artery territory formed after prior use of hemodialysis catheters. Considering the potential development period of the pseudoaneurysm related to prolonged use of these large caliber catheters, we opted for a surgical approach in our treatment strategy after unsuccessful ultrasound compression. Surgical intervention is considered an option for large pseudoaneurysms without a short neck or those that do not respond to other forms of treatment.^28^

In a review by Shah et al. (2004)^29^, it was demonstrated that surgical removal is more effective and safer for larger caliber catheters implanted in arteries of the cervical region. Meanwhile, Pikwer et al. (2009)^25^ showed that endovascular techniques can be used for the treatment of devices inadvertently implanted in the common carotid artery, including covered stents and a suture-mediated closure system device. At the HU-UEL, all patients were treated with emergency surgical cervical exploration.

The subclavian artery can also be treated using covered stents or hybrid surgery, involving endovascular balloon control of bleeding followed by open repair.^25^ Use of a specific suture-mediated arterial closure device has been shown to be effective in subclavian artery injuries.^30-32^ There is a reported case in which the vertebral artery ostium was occluded with a stent with no acute harm to the patient.^33^ Nevertheless, there is a potential risk that covering the origin of the vertebral artery could lead to basilar artery thrombosis with subsequent stroke. This, however, has not been documented following axillo-subclavian arterial trauma.^34^ When coverage of the vertebral artery ostium is necessary, it is recommended to document the patency of the contralateral vertebral artery with arteriography or CT angiography.^35^

Case reports of arteriovenous fistula after venous catheter implantation are also found in the literature.^36,37^ In a prospective cohort, there was a 0.86% incidence of arteriovenous fistula after catheterization for cardiological procedures,^38^ with a recommendation for non-surgical management and clinical and ultrasound follow-up, resolving one-third of fistulas within a year. In our single case of arteriovenous fistula, we opted for surgical treatment due to the pediatric patient’s long-term complication risks and low preoperative risk. In another case reported in the J Vasc Bras., there is a rare report of arteriovenous fistula and acute limb ischemia following hemodialysis catheter implantation in the inguinal region, treated with thrombectomy and surgical correction.^39^

One of the limitations of this study is the lack of data on ultrasound-guided puncture, which is available at our service but is not used as a routine protocol by other specialties. During the pandemic, there was an overload of severely ill patients, the healthcare team burden was excessive, and many less experienced doctors cared for patients. We believe that ultrasound use could reduce the rate of severe complications, especially for cases with unfavorable anatomy. Ultrasound guidance is recommended as the preferred method for vascular cannulation due to its superior safety and effectiveness, supported by evidence-based recommendations.^40^ It has been proposed that catheters suspected of inadvertent arterial implantation with a diameter greater than or equal to 7F should not be removed without evaluation by a vascular surgeon.^24^ Finally, it is considered possible that some patients with complications resulting from vascular devices may have died or been transferred before consulting with a specialist and would thus not have been identified in the present study.

## CONCLUSION

The pandemic brought new challenges for all specialties, and vascular surgery was no exception. Despite the increased number of severely ill patients, most patients in our service who underwent interventional procedures for vascular complications related to device implantation or use did not have a confirmed diagnosis of COVID-19, which contradicts emerging literature on the topic. Regardless of the large number of device implantations, exhaustion, and the presumed inexperience of COVID-19 care teams, the rate of severe vascular injuries that needed vascular intervention was similar to that described in pre-pandemic literature. It is essential for the vascular and endovascular surgery team to be prepared to manage complications associated with arterial and venous vascular device use.
